# Impact of the COVID-19 Pandemic on Habilitating Residential Communities for Unaccompanied Minors during the First Lockdown in Italy: The Educators’ Relational Perspective

**DOI:** 10.3390/ijerph18116166

**Published:** 2021-06-07

**Authors:** Sara Isernia, Francesca Sangiuliano Intra, Camilla Bussandri, Mario Clerici, Valeria Blasi, Francesca Baglio

**Affiliations:** 1 IRCCS Fondazione Don Carlo Gnocchi ONLUS, 20148 Milan, Italy; sisernia@dongnocchi.it (S.I.); mario.clerici@unimi.it (M.C.); vblasi@dongnocchi.it (V.B.); fbaglio@dongnocchi.it (F.B.); 2 Faculty of Education, Free University of Bozen-Bolzano, 39100 Bolzano, Italy; 3 Department of Physiopathology and Transplantation, University of Milan, 20122 Milan, Italy; camilla.bussandri@studenti.unimi.it

**Keywords:** COVID-19, pandemic, children, education, rehabilitation

## Abstract

(1) Background: Italian residential communities for unaccompanied minors suffered a long period of closure during the SARS-COV2 lockdown. Professional educators who work inside these institutions with the aim to habilitate children toward life-span achievements faced a great challenge and responsibility during this period. In this context, the psychological well-being and development of unaccompanied children were at high risk. The aim of this study was to investigate the impact of the lockdown on children living in residential communities from the educators’ perspective and to explore whether the educators’ relational lens was related to their perception and sense-making. (2) Methods: We conducted a mix-method study enrolling 21 educators in 10 residential communities who completed an interview and a self-construal scale. (3) Results: The interview was analyzed by a qualitative content method revealing 10 themes (social relationships, stand-by, emotions, new activities, new norms acceptance, end of lockdown, time, space, resilience, and achievements). Moreover, correlation analyses were performed to test the possible association between RISC and themes that emerged from the interviews, showing significant associations with four interview themes. (4) Conclusions: Our study highlights considerable lockdown effects on residential communities and the importance of educators’ relational approach, a tool for habilitating children and a protective factor against emotional overwhelming.

## 1. Introduction

Unaccompanied minors represent a highly vulnerable population due to the plethora of challenges and adverse lived experiences such as the loss of loved ones, departure from the country of origin, separation from parents, and poverty that lead them to be at high risk of relational and environmental difficulties [1]. In Italy, habilitating residential communities aim to provide their members with the possibility to achieve relational and educational needs and render them ready for full integration into society when coming of age.

In 2020, minors living in residential communities for unaccompanied children faced an enormous challenge due to the outbreak of the COVID-19 pandemic and its social consequences. From December 2019, the new SARS-COV2 virus manifested in Wuhan (China) as atypical pneumonia and then spread globally [2], with a considerable number of cases in Italy by the end of February 2020 [3]. The most critical effect of the pandemic consisted of the unpreparedness of the National Health Care System for the event [4]. Although the pandemic marginally affected minors in terms of the severity of epidemiological manifestations, with 2.1% of incidence and 0.2% of mortality under 18 years of age [5], the containment measures affected this population. New policies included physical distancing, lockdown, and closure of educational institutes, as well as a stand-by of support programs for youths and all health services except for the ones dedicated to COVID-19 recovery [6], with psychosocial, economic, and health-related impacts [7,8]. The psychosocial burden of the pandemic is indeed already having critical repercussions on children’s and adolescents’ mental health, with a still-underestimated and partly unknown magnitude [9,10], to such an extent that Cardenas et al. [11] named it a “parallel pandemic”. High rates of depression and anxiety are among the psycho-behavioral effects of perceived isolation in minors during pandemic lockdown [9,12,13]. In this context, children living in demanding situations could experience many other adverse effects [14,15,16,17]. In fact, as highlighted in recent studies which adopted a qualitative approach, the wide range of negative pandemic effects can be perceived as long-term changes in daily life, leading to a mental health deterioration [18]. This is true especially for vulnerable populations such as children and adolescents, and those with atypical development, such as autism spectrum disorder [19]. A paucity of evidence regarding these indirect effects of the virus on disadvantaged children, such as unaccompanied minors, needs to be overcome.

Early in the first pandemic phases, Italy has temporarily overtaken the official number of deaths of China (from 11 to 19 March 2020). The epidemic crisis was contained by highly restrictive lockdown measures starting with phase I from 9 March to 3 May, followed by a phase II until 14 June in which the lockdown measures were mitigated [4]. The phase I lockdown resulted in an interruption of most educational projects in the residential communities for children. In several cases, professional educators remained locked inside the residential structures with children, and had to manage and guide new routines, with a preferential and unique view of minors’ reactions to the emergency and responsibility in the containment of children’s emotional experience. This situation was of course at a high risk of adverse effects on the psychological well-being and development of unaccompanied children. 

Professionals who educate minors inside these institutions have a large responsibility by building relationships with children and being able to habilitate them towards life-span achievements in the absence of parenting scaffolding. In this context, the educator–child relational space represents a safe environment in which they together interiorize and manipulate emotions for sense-making experiences. Therefore, the relational lens of educators is a crucial vehicle through which the developmental process is triggered. It is indeed the dynamic relational space between the educator and the child that makes it possible to face challenges and adverse life experiences. The quality of the early relationships between children and their caregivers impacts how they manage their future relational responses, starting from how they can reach their developmental goals [20]. The relevance of adaptive and responsive relationships in the child’s development is widely supported by all the main psychological theories [21]. The other side of the relationships’ importance for individuals lies in their role for Self-definition. While some social psychological theories state that the Self is shaped based on abstract individual concepts [22], there are pieces of evidence that highlight how the relationships are the pivotal element through which individuals define, enhance, and express their Self [23,24]. In this latter case, the scientific literature speaks of Relational Interdependent Self-Construal (RISC), defined as a tendency to describe one’s own Self in terms of close relationships with others [25]. Such personal Self-definition influences how people behave within social sharing [26]. Having a high interdependent relational self-construal could suggest the attribution of a higher value to the relationships themselves [27]. Granting the definition of RISC and the particular characteristic of professional educators’ jobs, we hypothesized that their RISC level could impact their perception and sense-making related to the effect of the COVID-19 pandemic on unaccompanied minors.

Considering the large direct and indirect effects of the pandemic on social relationships at all levels, it is highly relevant to investigate its effect on the vulnerable population of unaccompanied minors living in residential structures. Unfortunately, there is little evidence on the psychological impacts of the coronavirus pandemic [28] and even less concerning its repercussions on unaccompanied children’s well-being. 

Stemming from these considerations, we aimed to explore the impact of the coronavirus pandemic on minors living in residential communities from the perspective of the educators and the relation with their self-construal. Moreover, we aimed at shifting from the “what” people are experiencing to the “how” they are experiencing it to fill the distance between the epidemiological and social–behavior models [29]. For this purpose, we conducted a mix-method, qualitative and quantitative research with a semi-structured interview and a quantitative scale of the RISC to identify the main perceived changes in the minors lives within the community and their association with the relational dimension of the Self.

Due to the psychological burden of the COVID-19 pandemic, it is reasonable to predict that it had a considerably large negative impact on more vulnerable populations [30,31]. The investigation of the impact on unaccompanied minors is thus warranted in order to develop appropriate interventions aimed at preventing maladaptive life outcomes.

## 2. Materials and Methods

### 2.1. Participants

The project involved 21 professional educators employed in 10 residential structures for unaccompanied minors of Milan and Parma (Northern Italy). The mission was to habilitate frailty Italian and foreign minors’ autonomy through individualized educational projects. After making contact with the institutions, we began the enrollment procedure. Inclusion criteria to recruit professionals were: (1) being employed in the residential structures, (2) working inside the institution during the COVID-19 pandemic, and (3) a Bachelor’s degree in “Health Professional Educator”. Before taking part in the study, each participant could clarify any doubts by inquiring the researcher and were provided written informed consent. All subjects gave consent for audio recording. The study was approved by the Review Board of the University of Milan (code number 20/20/cdl/AR/cb).

### 2.2. Procedure

A concurrent mix-method (qualitative plus quantitative) study was performed [32]. Subjects who voluntarily participated in the study were invited to attend an individual semi-structured interview via a telepresence online platform on the COVID-19 impact on the residents’ autonomy habilitation. Then, they were asked to complete a self-administered scale on Self-construal via an online survey. Globally, health professionals required 1 h of time availability for the study. The research was conducted between June and September 2020, after Phase II.

### 2.3. Materials

The study included a semi-structured interview to investigating participants’ perceived change inside the residential structure related to the period of the Phase I lockdown restrictions for the COVID-19 pandemic. Educators were asked to think about the changes related to the COVID-19 pandemic with respect to what they noticed inside the residential structures, and in particular, how their role and their job activities changed and how children experienced these changes. Furthermore, to measure the level of relational Self in educators, a self-report scale on Self-Construal was administered, the RISC [25]. This scale is composed of 11 items investigating the level of definition of the Self in terms of relationships with others (e.g., “My close relationships are an important reflection of who I am”), and participants are required to rate them by using a 7-point Likert scale. 

### 2.4. Mix-Method Analysis

#### 2.4.1. Qualitative Analysis

A category-based qualitative content analysis was performed to analyze semi-structured interviews by two researchers, adopting a data-driven approach. The analysis followed different steps. (a) Transcription: audio recordings were listened to by one researcher and then transcribed verbatim. (b) Identification of coding units/categories: after a thorough familiarization with the interviews’ contents, coding frames were individualized separately by two researchers in a double-blind modality. The agreement between the two researchers was then calculated (Cohen k) to assure the coders’ reliability. Categories identified from only one researcher were discussed and collegially resolved (including or deleting the category from the list). (c) Segmentation of text by coding units: text excerpts associated with each category, utilized in this step as coding frames, were highlighted and ordered in a qualitative matrix. (d) Computation of frequency and weight of the coding units: two indexes were computed: (1) the frequency of each code (by dividing the number of individuals who mentioned the category by the total number of participants), and (2) the weight of the code on the whole interview for each participant (by dividing the number of words associated to the different codes by the total words included in the interview transcription). (e) Keyword identification: keywords mentioned by the participants were extracted for each category by the interviews. (f) Meaning identification: different topics from each category were identified.

#### 2.4.2. Quantitative Analysis

Jamovi (Version 1.2; 2020) was used for the quantitative statistical analysis. A Shapiro–Wilk test was utilized to test the normality distribution of variables. Summary statistics were run to describe demographic characteristics. Pearson correlations were performed between the RISC scale and the weight of interview categories.

## 3. Results

In total, 10 residential communities for unaccompanied minors participated in the study (six in Milan and four in Parma—Northern Italy). The 21 health professionals who took part in the study, 14 females, had a mean age of 35 ± 9.43, and 8.33 ± 6.26 mean years of work experience. All participants completed the RISC scales, while one subject did not attend the interview session and was not considered for the qualitative analysis.

### 3.1. The Interview Results 

The double-blind identification of significant coding frames in the interview resulted in 10 (coder one) versus 12 (coder two) themes being highlighted. The agreement between coders was substantial (Cohen k = 0.74, percentage of agreement = 87%). The two categories found by only one coder were discussed and two of them were collegially excluded. 

The final significant categories identified were the following:

(1) Stand-by: educators reported the perception of an interruption, an abrupt stand-by, and/or a slowing down in different aspects of life inside the residential communities, especially outdoor educational activities dedicated to autonomy achievements.
(2) Emotions: the main emotional experiences perceived were related to disruptive emotions such as fear and/or suffering and the difficulty of the educators in containing the disruptive emotions of the children.
(3) Social Relationships: perceived changes in social relationships and social life inside and outside the residential community were described and the role of social relationships in coping with the lockdown was highlighted as a key factor.
(4) Space: educators reported changes in the conception of the space, related to both the reduction of the physical space and the overlapping between this and the relational space.
(5) Time: according to changes in the conception of the space, a similar new interpretation of the time dimension occurred in terms of both an opportunity to take her/his own time, but also the uncomfortable sense of emptiness and the necessity to fill it.
(6) Reorganization of daily routines: since the community was locked down, the need both to reschedule the daily routine and to introduce new activities occurred.
(7) New norms acceptance: educators highlighted the minors’ difficulties in the understanding of the causes underlying the lockdown and the new rules for virus containment.
(8) Resilience: the appearance of resilient behaviors was described in terms of adaptive strategies in order to cope with a new normal.
(9) Achievements: educators reported acquisitions and developments specifically related to new skills during the lockdown period.
(10) Lockdown end: participants referred to their reflections on the difficulties that children were likely to face after the end of the lockdown period when all activities were going to restart.

Globally, the category that most of the participants (85%) focused on was *social relationships*, followed by *stand-by* (80%). About 45% of educators deepened *emotions*, *new norms acceptance*, *reorganization of daily routine*, and *lockdown end* categories. Thirty-five percent of participants dedicated part of their answers to *time*, *space,* and *resilience* categories. Finally, only a few individuals addressed the topic *achievements (16%)*. Table 1 summarizes the frequency of each category, the mean weight of the category (the portion of the text of the answer dedicated to the specific code), the explicit keywords, and the meaning associated with each category.

### 3.2. Mix-Method Results

Correlations were performed between RISC and code weights to test the relation between self-construal and the sense-making related to changes during the COVID-19 pandemic inside the residence (see Table 2).

**Figure 1:**
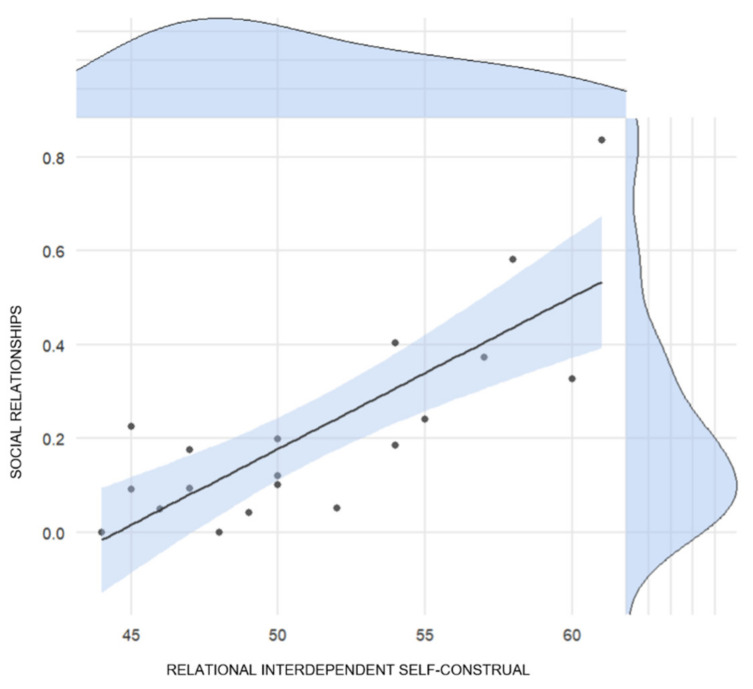
Correlation between the weight of the social relationship category of the qualitative interview and the Relational Interdependent Self-Construal (RISC) score.

Significant associations between RISC and Social Relationships (direct association, *p* < 0.001; see Figure 1), Emotions (inverse association, *p* = 0.007), Space (direct association, *p* = 0.033), and Lockdown End (inverse association, *p* = 0.007) were found.

## 4. Discussion

The present work reported a mix-method study aimed at exploring the COVID-19 pandemic experience in Italian residential communities for unaccompanied minors by interviewing the educators that lived inside the community with the children during Spring and Summer 2020. In this whole period in Italy, within the communities, a very strict lockdown was carried out with several restrictions, such as the impossibility to perform any activity outside the community and the children were prevented from visiting their relatives. The qualitative analysis of the semi-structured interview revealed several recurring themes related to broad-spectrum changes in the communities’ life with a central role of social relationships. Moreover, in agreement with this result, the quantitative analysis highlighted an interesting correlation between the level of educators’ RISC and the weight attributed to changes in social relationships, emotion, space, and the perception of the lockdown end.

The general frame in which most of the interview’s responses were allocated was the feeling of stand-by, a concept highlighted by 80% of the educators. Significant repercussions of such a slow-down were community discharges blocked (“All the discharges slowed down, we all are in stand-by”), interruption of educational activities outside the residential communities (“Movement autonomy outside of the center had to be interrupted”), such as going autonomously to school, with a direct impact of the children’s life plans perceived as being blocked as well.

The most frequent theme highlighted by educators was “social relationships”, which came up in 85% of the interviews, both in terms of negative changes and positive resources. As contact with the external environment became less frequent, social relationships inside the residential community became more intimate with the residents’ perception as an in-group with a shared identity in facing the pandemic emergency (“We have to survive together”). On the other hand, the ambivalence of the role of community social relationships during lockdown emerged: peers’ interactions within the community members increased, probably offering a protective factor against depression [33], but at the same time, children often chose to spend time alone in their own room or use their smartphone, suggesting the necessity to find a private space. The educators’ closeness strengthened as the unique vehicle of educational achievements, but this proximity was often perceived as a forced compresence (“We were forced living together”). These controversial aspects mirrored the COVID-19 responses identified by Bavel et al. [10]: elevating the in-group and perceiving a shared social identity versus a sense of forced proximity with the immediate loved ones in lockdown. 

Interestingly, changes in social relationships fostered a social redefinition of space. The “space” category appeared in the 35% of the interviews and was perceived as a potential relational space and consequently managed: the adolescents’ room became a salon for social activities or a space to be alienated from the group (“Being alienated from the group, alone in their own room”, “Utilizing their bedroom as living room to stay in groups”). Educators observed a transformation of the physical space’s conception, reshaped and resized according to the miniaturized living habitat. In contrast with the shrinking space, time was perceived as expanded as activities stopped because of the lockdown. Time, as an interview theme, was highlighted by 37% of the participants, in particular regarding the need to manage it. The associated meanings, again, were ambivalent: a threatening time to be filled to prevent a sense of emptiness (“Schedules became more difficult to organize”), or a favorable time, an opportunity to focus on activities for which, usually, there is not enough time (“The opportunity of an extended time…days free from all commitments”). In accordance with this theme, the need to reorganize the daily schedule of activities was identified as another category in the interview, as reflected in 45% of the responses. Meanings emerged were referred to both the practical activities that were possible to do and, more in general, the educational goals they were working towards (“The educational work is changed”). 

Another significant theme found in our data (45% of the educators) was the enormous impact of the COVID-19 pandemic consequences on the emotional sphere affecting both educators and minors, as described in previous reports [34]. The emotions more frequently reported were apathy, fear, sadness, fragility, suffering, anxiety, insecurity, exasperation, trauma, boredom, and frustration, suggesting a disrupting prevalence of negative affect. However, one of the most interesting points was the role of negative emotions, according to the educators’ perspective, that was perceived as complex to contain or manage (“The management of emotions was the most difficult issue for them”), but also, in some cases, relevant for promoting behaviors directed towards children’ physical and mental health protection (“By entrusting them, they felt supported”). The identification of the theme related to emotions is not surprising, considering the strict interdependence of emotional responding and social relationships [35,36,37,38]. Besides the impact of negative emotions, educators reported an emotional flattening in the children (“The new reality flattened them”), that can be interpreted as a response to the lack of experiences due to the confinement and/or as a protective defensive mechanism against the negative emotions [39]. The emergence of negative emotions was not perceived only as a problem but also as a positive developmental pressure towards coping mechanisms. In fact, in this context, 37% of the educators observed resilient behaviors in response to such negative emotions as the initiative of group activities in decorating the common rooms and group games (“It is as if they found a new normality”). Interestingly, the educators detected a link between resilient behaviors and the acceptance of the new norms, another category that was reported in 45% of the interviews, by comparing those children who understood them (“It is as if they were following new rules for a new normal way of living in the community”) and those who did not (“They do not comprehend the reason why they cannot go outside”). Educators observed that adaptive mechanisms took place only if the comprehension of the new norms by the children occurred. Accordingly, and even more interestingly, they discovered new skills that were valuable for facing the social and emotional deprivation stemming from the lockdown norms, such as achieving new autonomies. These new achievements and autonomies have been summarized as another theme highlighted by 16% of educators. These skills allowed them to relocate themselves to what has been called a “new normality”. A relevant example of this dynamic is e-learning, pictured as a serious challenge that unaccompanied children had to face in the residential communities during the lockdown. Different from conventional situations, in which distant modality of learning management fell on parents, minors in the residential communities had to arrange school through online platforms without their assistance, even coping with an economically disadvantaged condition. In fact, as has been noted by others [40,41], distance learning deconstructed the key principles of democratic, inclusive, and embodied teaching. Unexpectedly, in our case, minors showed reaching full autonomy in managing distance learning and in achieving the required skills (“They were totally autonomous in the management of remote learning”). Additionally, contextual requests of support to accomplish learning goals were spontaneous (“Children themselves requested to receive additional teaching of the Italian language by volunteers”). This report showed evidence of unaccompanied minors’ functional response to pandemic lockdown with adequate coping strategies, depicting them, in line with the literature, as active survivors [1]. 

The new normality was also recognized and experienced by the professional figure of the educator. By reflecting on their role in the community, educators felt their activity mutated to accomplish children’s changing needs in that historical moment (“The educational work is changed). In particular, the impossibility for all non-residents (e.g., parents and psychologists) to enter the community exposed the educators to the necessity to partially compensate for these absences with a substantial change in roles and rules: educators were the only reference point for minors in the community and, for example, they needed to replace, together with the children, the roles of housekeeper and cook. Importantly, the situation of the perceived emergency was described as continuing after the lockdown. In fact, portions of the responses (45% of the educators) were dedicated to the perception of the end of the lockdown, in terms of a situation of a slow re-starting of activities, linked to the fear of a relapsing of the pandemic. Restrictions determined the impossibility to perform several activities supporting children’s autonomies, such as using public transportation by themselves to go visit their parents. The educators perceived that this loss of autonomy resulted in a sort of “regression” in the adolescents as if they had to re-learn several skills after the lockdown ended, and that children needed support to rebuild their life projects (“The regression is still ongoing and we are hardly trying to pick up the pieces”, “There is still the handbrake on”) [34].

By testing the potential influence of educators’ RISC on their sense-making process of the COVID-19 lockdown, we found a significant correlation between these two components. In fact, educators with a high relational perspective deepened to a greater degree the consequences on social relationships of the lockdown, suggesting the adoption of a social lens. Additionally, they tended to report changes in the conception of the space in its dual meaning, in terms of both physical and relational–social space. Noteworthy, the negative correlation between the RISC and the weight of the interview emotions category suggests that the relational lens could be a protective factor: the educators with a high relational perspective discussed less the negative emotional impact of the pandemic. Moreover, the level of RISC was inversely associated with the negative view of the post-lockdown period, likely suggesting that a relational view allowed the educators to maintain a more optimistic representation of the immediate future. The framework of the analytic field theory [42,43,44] can support the interpretation of these results. According to this model, the interdependence between the context and the relationship between two people is mediated by the capacity of the relational couple to construct metaphors and meanings of the joint experience. This latter represents an element that does not depend on the single person’s emotions but belongs to a third-party element, the analytic field, with a great transformative potential [45]. Assuming this perspective, in the relationship between the educator and the child, what is experienced in any given moment can be attributed to how the couple is interacting and not to the single person’s feelings. In this case, it is the relationship between the two that creates a relational space in which it is possible to modify the emotional impact of the pandemic with a consequent more optimistic view. 

Furthermore, the importance of the relational approach of caregivers on the emotional, social, and psychological adjustment of the child has been demonstrated in previous works [46,47,48], and in different contexts. Thrasher and Grossmann [48], by studying the role of quality child–caregiver relationship level, demonstrated that a high relationship quality supports a child’s emotional recognition and social competencies. Moreover, emotion socialization parenting programs are effective in the development of emotion regulation in young children [49]. Additionally, considering the relationship between child and educator, different contributions demonstrated that the quality of this relationship is a key factor for a minor’s school outcome, social skills, and behavior [50,51,52]. Globally, when emotional support is provided by educators to the child, developmental outcomes were better achieved [53].

This work presents some limitations. Our sample of educators was small and the results have to be interpreted with caution. However, our goal was to explore the impacts of the COVID-19 pandemic and lockdown period on the residential communities, and we involved 10 institutions in Northern Italy, hosting in total a considerable group of children. Moreover, we did not consider in our analysis separate occurrences inside each structure during the pandemic, such as the number of contagions, which could have affected the reported experience.

## 5. Conclusions

To conclude, our study is relevant in its attempt to explore the COVID-19 pandemic’s impact on unaccompanied minor’s communities in residential structures. Significant effects of the lockdown on social relationships were found and a positive influence of the relational perspective of educators has been highlighted as a potential protective resource against the negative emotional impact of the pandemic. Our results indicate a possible way for professional educators to maintain their pivotal role in habilitating children for their developmental achievements without being overwhelmed by the emotional impact of the traumatic experiences that can occur, particularly when working with vulnerable and fragile populations.

## Figures and Tables

**Table 1:** A summary of the results of the qualitative analysis.

Frequency	Weight	Code	Examples	Explicit Keywords	Meanings
85%	23%	Social relationships	“They came together as a group…the community is more like a family”, “We have to survive together”, “Separate himself from others or separate himself together with others”	Relational sphere, role model, being cared for, trust, being in a group, isolation	Loneliness, new relational autonomies, emotional meaning, forced living together
80%	28%	Stand-by	“All the discharges slowed down, we all are in stand-by”, “Movement autonomy outside of the center had to be interrupted”, “Life plan blocked”	Slowing down, stand-by, waiting, interruption, regression, withdrawal, rescheduling, postponing	Sense of uselessness, fragility, unpredictability, insecurity
45%	18%	Emotions	“The new reality flattened them”, “The management of emotions was the most difficult issue for them”, “By entrusting them, they felt supported”	Apathy, fear, emotional sphere, emotions regulation, sadness, fragility, trust, suffering, anxiety, insecurity, exasperation, trauma, boredom, frustration	Pervasiveness of emotional aspects, emotional burden
47%	22%	New norms acceptance	“They do not comprehend the reason why they cannot go outside”, “They understood the situation and followed the rules”, “It is as if they have found a new normal way of living”	Internalization of the rules, comprehension of the situation, historical contextualization, adaptation to the situation	Only through comprehension can adaptation be reached, otherwise the internalization of the norms is not generalized
45%	29%	Reorganization of daily routines	“The educational work is changed”, “It has been a suspended moment when it was all fine”, “It is like we were in a COVID ward”.	*No Keywords identified*	Concrete change in individual roles
45%	27%	End of lockdown	“The regression is still ongoing and we are hardly trying to pick up the pieces”, “The companies have mobilized resources to restart internships”, “There is still the handbrake on”	Regression, re-learning, restarting, reconstruction	End that is not an end
37%	18%	Time	“18 youths 24 h/7 d”, “Schedules became more difficult to organize”, “The opportunity of an extended time…days free from all commitments”	24/7, an expanded time, respected schedule, different time schedule than usual	Time as an opportunity/resource but also time to fill
37%	14%	Resilience	“Surviving together throughout this period”, It is as if they found a new normal”, “They stayed closed in their room and they attended lessons in autonomy”	New normal	Adaptation, changes in priority, reallocation
35%	24%	Space	“Their external space is also a space for decompressing”, “Being alienated from the group, alone in their own room”, “Utilizing their bedroom as living room to stay in groups”.	Shutting out the rest of the world, decompression space, external space, isolation, distance, external world, living space, resizing	Redefining the space, physical space that becomes a space for relationships
16%	31%	Achievements	“They were totally autonomous in the management of remote learning”, “Autonomy in the organization of games”, “Children themselves requested to receive additional teaching of the Italian language by volunteers”	Autonomy	Contextual request for support

**Table 2:** Results of correlations between interview categories’ weight and Relational Interdependent Self-Construal (RISC) score.

Interview Categories	RISC	
	r	*p*
Social Relationships	0.732	<0.001
Stand-by	−0.124	0.601
Emotions	−0.579	0.007
Space	0.479	0.033
Time	0.108	0.650
Reorganization of daily routines	0.094	0.692
New norms acceptance	−0.312	0.160
Resilience	−0.013	0.957
Achievements	0.011	0.964
Lockdown end	−0.581	0.007

## Data Availability

The data presented in this study are available on request from the corresponding author. The data are not publicly available due to privacy issues.
